# The retina rapidly incorporates ingested C20-D_3_-vitamin A in a swine model

**Published:** 2013-07-25

**Authors:** Doina M. Mihai, Hongfeng Jiang, William S. Blaner, Alexander Romanov, Ilyas Washington

**Affiliations:** 1 Department of Ophthalmology, Columbia University Medical Center New York, NY; 2 Department of Medicine, Columbia University Medical Center, New York, NY; 3 Institute of Comparative Medicine, Columbia University Medical Center, New York, NY

## Abstract

**Purpose:**

To determine how the retina uses vitamin A for vision, we studied the flux of oral vitamin A into and out of the swine retina.

**Methods:**

We administered labeled vitamin A to swine daily for 30 days and measured the percent of the labeled vitamin A to native unlabeled vitamin A in the retinal epithelium, neuroretina, plasma, liver, lung, and kidney.

**Results:**

We show that during normal vitamin A homeostasis, the retina rapidly assimilates newly ingested dietary vitamin A, which replaces native vitamin A. Retinal vitamin A is turned over faster than previously thought. Provitamin A carotenoids do not significantly contribute to retinal vitamin A pools when consuming diets adequate in vitamin A.

**Conclusions:**

Fast vitamin A turnover in the retina has direct implications for emerging therapies to prevent major forms of blindness based on controlling the concentrations of retinal vitamin A.

## Introduction

During vision, in the disc lumen of the normal functioning eye, vitamin A as retinaldehyde becomes bound to a phosphatidylethanolamine lipid to form a retinaldehyde–phosphatidylethanolamine complex. A rim protein is responsible for transporting this complex out of the disc lumen [1]. Patients with Stargardt disease have a genetic defect in the gene encoding for this rim protein, the ATP-binding cassette sub-family A gene [2]. With a dysfunctioning transporter, the vitamin A complex reacts with vitamin A aldehyde to form vitamin A dimers, N-retinylidene-N-retinylethanolamine (A2E) and all-trans-retinal dimer (ATR-dimer), which precede clinical signs of Stargardt. Vitamin A dimers are also elevated in the eyes of those with age-related macular degeneration and Best disease.

The observations that the accumulation of vitamin A dimers precede retinal degeneration and that these dimers can cause cellular and retinal toxicity in animal and cell models have prompted the development of pharmacological interventions to retard vitamin A dimerization [3-7]. We have shown that replacing the eye’s natural vitamin A with vitamin A deuterated at carbon-20 (C20-D_3_-vitamin A) prevents the formation of vitamin A dimers [8,9]. However, an intervention based on controlling the concentration of retinal vitamin A has been questioned because 1) the retina is notoriously hard to deplete of vitamin A [10,11]; 2) vitamin A has a long half-life in the body of 140 days [12]; and 3) the many dietary sources of vitamin A and provitamin A carotenoids make it hard to limit intake of dietary vitamin A. Accordingly, we followed the rates of C20-D_3_-vitamin A accumulation in the retinal epithelium (RE) and the neuroretina of the eye, and in the plasma, liver, lung, and kidney of swine given a C20-D_3_-vitamin A capsule while fed normal amounts of dietary provitamin A carotenoids. We found that under normal vitamin A homeostasis, retinal vitamin A is rapidly replaced with newly ingested vitamin A, and that plasma and retinal vitamin A pools are in equilibrium. These data suggest that C20-D_3_-vitamin A can rapidly replace vitamin A in the retina to potentially prevent vitamin A dimerization, making pharmacological control of retinal vitamin A a pragmatic clinical approach to combat major forms of retinal degeneration.

## Methods

### Animals

Columbia University’s Animal Care and Use Committee approved this study. We used male swine, Yorkshire crossbreeds, 4 weeks of age, weighing 7 kg (Animal Biotech Industries, Inc., Danboro, PA).

### Intervention

Animals were given 3 mg of retinyl acetate per day as a mixture of 2.85 mg C20-D_3_-retinyl acetate (Alkeus Pharmaceuticals, Boston, MA) and 0.15 mg retinyl acetate (Sigma Aldrich, St. Louis, MO). The retinyl acetate mixture was dissolved in olive oil at a concentration of 3 mg mixture/200 μl olive oil. As a stabilizer, we added 3 mg of butylated hydroxytoluene per 200 μl of olive oil. The oil (200 μl) was then injected into gelatin capsules, which were inserted into cherry tomatoes and given daily to each animal during their morning feeding.

Five treated animals were housed per cage. In each cage, in the morning, we placed 3 kg per day of a diet without supplemented or naturally derived preformed vitamin A. The diet consisted of ground corn, wheat middlings, dehulled soybean meal, porcine meat meal, cane molasses, calcium carbonate, salt, sodium selenite, biotin, trace mineral premix, choline chloride, folic acid, cholecalciferol (vitamin D-3), zinc oxide, dicalcium phosphate, dl-alpha tocopheryl acetate, calcium pantothenate, nicotinic acid, vitamin B-12, riboflavin, and L-lysine. As a source of carotenoids, the diet contained 10% dehydrated alfalfa. In the late afternoon, animals were given cracked corn (3 kg per day per cage) and a treat of peanut butter, tomatoes, apples, and/or bananas. After 2 weeks on this diet, animals weighed an average of 12±2 kg and at 4 weeks on this diet, animals weighed an average of 17±3 kg.

### Tissue collection

Treated animals were sacrificed with intravenous injection of 1ml Euthasol® (Virbac AH, Inc., Fort Worth, TX) per 10 lb of body weight, after 2 and 4 weeks of daily dosing and 24h after administration of their last vitamin A capsule. Eyes were removed under room lighting, immediately placed in a solution of PBS (Phosphate Buffered Saline, Catalog number 21-030-CV, Mediatech Inc., Manassas, VA), and kept in the dark for 2 h. Subsequently, under 660 nm light, eyes were placed in a 4% formaldehyde [13] PBS solution and refrigerated for three weeks in the dark. Eyes were dissected under room lighting. The neuroretina and RE were extracted and analyzed separately. Blood was collected in tubes containing EDTA. Plasma was separated from cells within 1 h by centrifugation and kept frozen at −80 °C until use. Other tissues (40–50 g) were collected within 20 min after death, washed free of blood with saline, and stored at −80 °C in saline until use.

### Tissue extraction

Samples were processed under a dim yellow light as previously described [14]. Tissues were homogenized in saline with a bead mill homogenizer (BBY24M Bullet Blender^®^ STORM, Next Advance Inc., Averill Park, NY) using stainless steel beads. Plasma or tissue homogenate was mixed with three volume equivalents of absolute ethanol, 100 pmol of retinyl acetate (added as internal standard), and three volume equivalents of hexane containing 1 mg butylated hydroxytoluene per milliliter. The samples were vortexed for 1 min on the highest setting and the phases separated by centrifugation at 3,000 g for 10 min. We then transferred the organic phase into a new tube and evaporated the organic solvent under a stream of nitrogen. Samples were immediately resuspended in 50 μl of methanol and transferred into liquid chromatography–mass spectrometry (LC-MS) vials (Waters, Milford, MA). Samples were maintained at 4 °C in an autosampler and 5 μl was loaded onto the LC-MS system.

### Retinoid analysis

The LC-MS system was a Waters Xevo TQ MS ACQUITY UPLC controlled by MassLynx Software v 4.1, attached to a Waters ACQUITY UPLC BEH phenyl column (3.0 mm inner diameter ×100 mm with 1.7 μm particles) and a 2.1×5 mm guard column with the same packing material (Waters). The column was held at 40 °C. The flow rate was 500 μl per minute and initiated with 5% phase A (water containing 0.2% formic acid and 1 mM ammonium formate) and 95% mobile phase B (methanol containing 0.2% formic acid and 1 mM ammonium formate). Mobile phase B was increased linearly to 97.5% over 5 min. The column was washed with 99% methanol for 1 min between every sample. Positive electrospray ionization MS with selected ion recording was performed using the following parameters: capillary voltage -4.0 kV, source temperature 150 °C, desolvation temperature 500 °C, and desolvation gas flow 1,000 l per hour. Multiple selected reaction monitoring transitions for retinoids were as follows: retinol and retinyl esters m/z 269.2, C20-D_3_-retinol and retinyl esters m/z 272.2, and retinaldehyde 285.2 and C20-D_3_- retinaldehyde 288.2. Difference species were identified by comparing the retention times of experimental compounds with those of authentic standards. Concentrations of retinol and retinyl esters in the plasma were quantitated by comparing integrated peak areas for those of each retinoid against those of known amounts of purified standards. Any potential loss during extraction was accounted for by adjusting for the recovery of the internal standard added before extraction.

Under our LC conditions, retinol eluted at 1.5 min, retinaldehyde at 1.7 min, and retinyl acetate at 1.8 min. Retinyl esters eluted as five peaks between 3.5 and 4.5 min. Retinyl myristoleate (C14:1), retinyl myristate (C14), retinyl oleate (C18:1), and retinyl stearate (C18) eluted separately, while retinyl palmitoleate (C16:1) co-eluted with retinyl alpha-linoleate (C18:3) and retinyl palmitate (C16) co-eluted with retinyl linoleate (C18:2). For negative controls, we analyzed tissues from control animals not given deuterated vitamin A (Cohen Max Insel Animal Organs & Tissues for Research Inc., Livingston, NJ). If ions at 272 and 288 mass units, with the same retention times, were detected in control samples, they were subtracted from the treated samples.

## Results

To determine how quickly retinal vitamin A pools exchange with newly ingested vitamin A, we administered C20-D_3_-retinyl acetate to young swine and measured the accumulation of the deuterated vitamin in the retina. To model the median dietary intake of unlabeled dietary vitamin A in humans, we administered, a capsule containing 3 mg vitamin A as 95% C20-D_3_-retinyl acetate and 5% unlabeled retinyl acetate. Animals were fed a vitamin A free diet but were given above the recommended daily allowance of provitamin A carotenoids. After 14 and 28 days of this treatment, we collected the retinal epithelium, neuroretina, plasma, liver, lung, and kidney and determined the percentage of C20-D_3_-vitamin A relative to total vitamin A in the tissues using LC-MS (Table 1).

**Table 1:** Uptake of newly ingested vitamin A into the retina.

**Tissue vitamin A**	**2-weeks**	**4-weeks**
**Neuroretina Retinaldehyde**	82±7%	94±2%
**Retinal Epithelium Esters**	70±4%	74±2%
**Retinal Epithelium Retinaldehyde**	77±7%	86±3%
**Plasma Retinol**	84±2%	86±3%
**Liver Esters**	57±6%	67±4%
**Liver Retinol**	62±6%	74±3%
**Kidney Esters**	68±9%	79±3%
**Kidney Retinol**	45±5%	42±13%

Percentage of deuterated vitamin A in selected tissues of swine given 3 mg of oral vitamin A as, 95% deuterated vitamin A and 5% vitamin A, daily for two and four weeks. Data represent an average of five animals per group per time point (n=5). Standard deviations are shown.

After two weeks of administration, 84±2% of plasma retinol was C20-D_3_-retinol. Plasma retinyl esters were all deuterated and represented 5% of plasma retinol. The neuroretina’s retinaldehyde pool was 82±7% C20-D_3_-retinaldehyde. The percent deuteration was equal to the percentage of deuterated retinol in the plasma. In the RE, 70±4% retinyl esters and 77±7% of retinaldehyde were deuterated. The kidney (45±5% for retinol and 68±9% for esters) and liver (62±6% for retinol and 57±6% for esters) were less enriched compared to the retina and plasma. The lung contained no detectable amounts of retinol; small amounts of retinyl esters were detected, and these were all deuterated.

At 4 weeks, plasma retinol was enriched further by 2% to 86±3%. The neuroretina’s deuterated vitamin A reached 94±2% and reflected the steady-state intake of the vitamin mixture, i.e., 95% deuterated vitamin A. Vitamin enrichment in the RE was close to that of the plasma (74±2% for retinyl esters and 86±3% for retinaldehyde). The liver was enriched approximately 10% more compared to 2 weeks of treatment (74±3% for retinol and 67±4% for esters). Kidney retinol remained the same (42±13%), but enrichment increased by 10% for kidney retinyl esters (79±3%). Enrichment in the lung remained the same as in the 2 week animals, containing only a small amount of deuterated esters (data not shown).

Total plasma vitamin A (retinol and retinyl esters) were the same in the 2-week and -4 week animals, consistent with homeostatic control [15]. Total liver vitamin A increased 21±17% after 4 weeks of treatment compared to animals treated for 2 weeks. All clinical blood parameters for the treated animals, alkaline phosphatase, gamma-glutamyltransferase, aspartate aminotransferase, alanine aminotransferase, amylase, blood urea nitrogen, glucose, phosphorus, calcium, albumin, cholesterol, uric acid, creatine kinase, creatinine, total bilirubin, total protein, globulin, blood urea nitrogen to creatinine ratio, albumin to globulin ratio, sodium, potassium, and chloride, were within the 95th percentile for healthy 8-week-old swine [16] (Table 2).

**Table 2:** Deuterated vitamin A showed no toxicity in response to up to four weeks daily dosing.

**Test**	**2-weeks**	**4-weeks**	**units**
**alkaline phosphatase**	234±50	252±40	U/l
**gamma-glutamyltransferase (GGT)**	52±20	69±31	U/l
**aspartate aminotransferase (AST)**	201±12	45±8	U/l
**alanine aminotransferase (ALT)**	50±28	39±8	U/l
**aSmylase**	1573±480	1565±93	U/l
**blood urea nitrogen (BUN)**	12±4	17±1	mg/dl
**Glucose**	137±69	108±16	mg/dl
**Phosphorus**	11±1	11±1	mg/dl
**calcium (less than)**	11±2	11±1	mg/dl
**albumin**	3.0±0.7	2.0±0.4	mg/dl
**cholesterol**	98±53	87±12	mg/dl
**uric acid (less than)**	1	1	mg/dl
**creatine kinase (CPK)**	779±274	463±71	mg/dl
**creatinine**	1.0±0.3	1.0±0.1	mg/dl
**total bilirubin (less than)**	0.06	0.06	mg/dl
**total protein**	5±1	5.0±0.2	g/dl
**globulin**	3.0±1	3.0±0.2	g/dl
**blood urea nitrogen to creatinine ratio**	20±2	32±4	-
**albumin to globulin ratio**	1.0±0.3	1.0±0.2	-
**sodium**	136±5	139±2	mmol/l
**potassium**	6±3	5.0±0.4	mmol/l
**chloride**	102±2	101±2	mmol/l

Data represent an average of five animals per group per time point (n=5). Standard deviations are shown.

### Statistics

Data represent an average of five animals per group per time point (n=5). Means and standard deviations are given. Comparisons between 2 and 4 weeks were made with two-tailed, paired t-tests. For all tissues except the kidney, the percent of enriched vitamin A at 4 weeks was significantly increased compared to the percent at 2 weeks, as defined by a p value of less than 0.05.

## Discussion

The successful translation of the use of C20-D_3_-vitamin A to prevent vitamin A dimerization in humans depends on attaining a high steady-state ratio of the deuterated vitamin over total vitamin A in the disc lumen of the neuroretina. To do this, the ingested ratio of deuterated vitamin A to vitamin A should be as high as possible, but the total amount of ingested vitamin A (nondeuterated and deuterated) should be under the recognized safe limits. In the United States, the median daily intake of vitamin A as retinol is 0.3 mg, which comes mainly from the consumption of vitamin A fortified milk, butter, margarine, egg yolks, and ready-to-eat cereals [17]. Thus, on prolonged consumption of 3 mg of C20-D_3_-vitamin A per day, 91% of the body’s vitamin A would eventually be replaced by C20-D_3_-vitamin A and the combined total amount of ingested vitamin A would still be well within safe limits [18]. If consumption of dietary, nondeuterated vitamin A were to be halved, 95% of the steady-state intake would be C20-D_3_-vitamin A. Here, swine were administered 95% C20-D_3_-vitamin A to 5% vitamin A. For the 7 to 17 kg swine used in this study, 3 mg of retinyl acetate corresponded to 5 to 8 times the recommended daily allowance of retinyl acetate (0.38–0.60 mg per day, NRC, 1998) and was below the toxic, chronic intake of vitamin A for swine [19].

**Figure 1:**
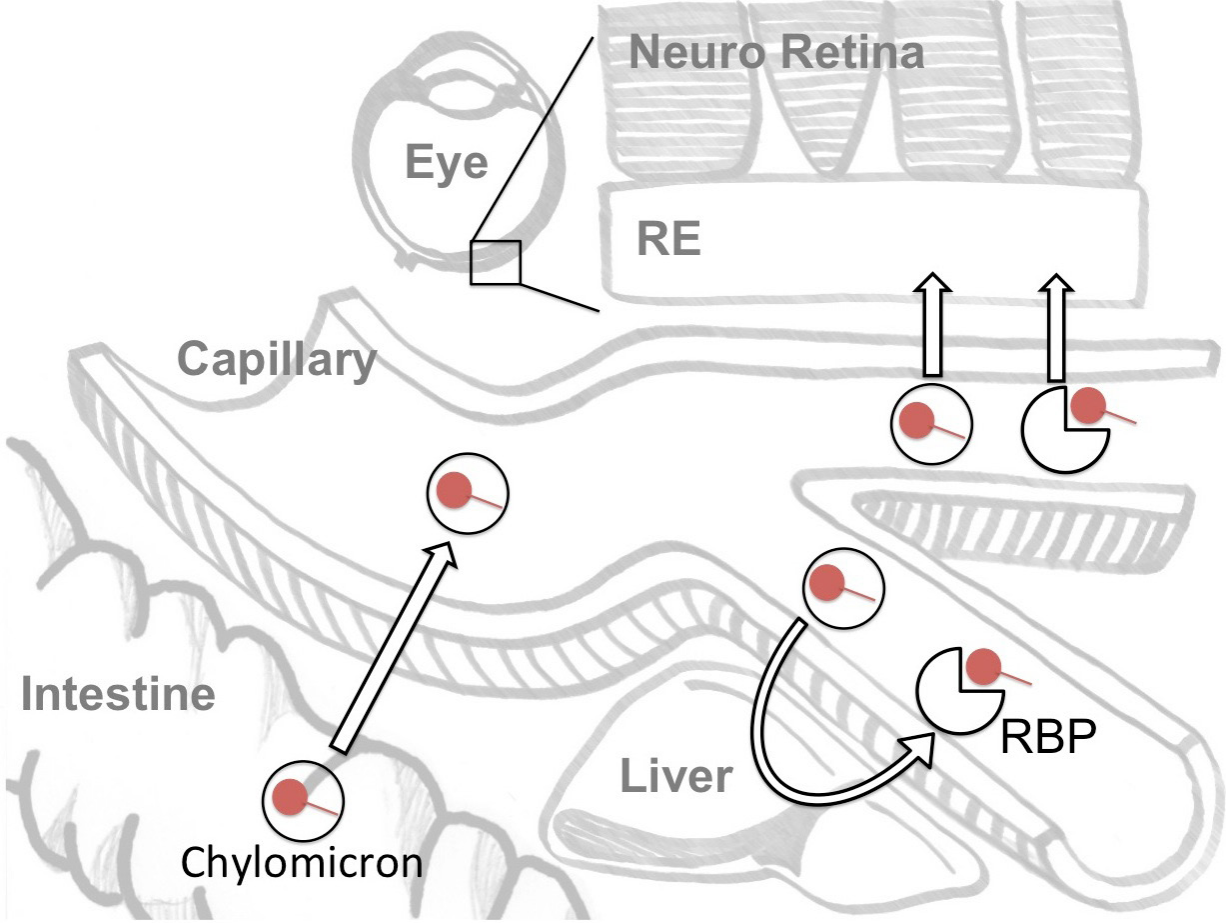
Vitamin A absorption and retina uptake Vitamin A is denoted as lollipops. RBP: Retinol binding protein. RE: retinal epithelium. Dietary vitamin A is absorbed by the intestine and packaged into chylomicrons, which are secreted into the circulation. Plasma chylomicrons can deliver vitamin A to tissues or to the liver where vitamin A is stored and rereleased into circulation as retinol bound to its binding protein (RBP) for tissue uptake.

In humans, absorption of vitamin A (as retinol, retinyl esters, or retinaldehyde) is nearly complete; at doses below 15 mg, 70 to 90% is absorbed by enterocytes in the small intestine within 2 to 6 h [20-22]. Enterocytes transform and package vitamin A into chylomicrons containing 90–95% retinyl esters and 5–10% retinol (Figure 1). The chylomicrons enter the general circulation where they can distribute vitamin A directly to the retina and other tissues or can be taken up by the liver. In the liver, the vitamin is stored, metabolized and/or is released, bound to retinol binding protein (RBP), into the general circulation for tissue uptake. Like the liver, the RE acts as a storage depot for vitamin A. The vitamin A pool in the RE is composed of mainly retinyl esters [23] and is used by the neuroretina to enable vision.

In this study, we used 4- to 8-week-old swine because they have similar 1) anatomic and physiologic gastrointestinal tracts to humans [24]; 2) similar vitamin A requirements to adult humans (0.38–0.60 mg of retinyl acetate per day) [24]; and 3) similar concentrations of plasma vitamin A (≈1.2 μM). For these and other reasons, swine are widely used to model vitamin A pharmacokinetics in humans [25-29].

Our data suggest that at normal vitamin A homeostasis, the vitamin A pool in the neuroretina quickly equilibrates with newly ingested vitamin A. This suggests that C20-D_3_-vitamin A can rapidly accumulate in the neuroretina to prevent the formation of vitamin A dimers. This finding is in contrast to the belief that the retina’s vitamin A pool is turned over very slowly. Previous studies, suggesting a long half-life of retinal vitamin A [10,11], were done in animal models during vitamin A deprivation and as such cannot necessarily be applied to animals consuming vitamin A adequate diets.

Data further suggest that retinal vitamin A quickly equilibrates with plasma vitamin A, revealing that plasma vitamin A can be used as a biomarker to predict the percentage of deuterated vitamin A in the retina. A fast plasma-retinal vitamin A equilibrium is consistent with 1) RPE cells and retinal blood vessels of the retina having some of the highest concentrations of RBP-receptors [30,31]; 2) the observation that the first symptom of depleting one’s diet of vitamin A, delayed dark adaptation, can occur within days, despite normal liver stores [32-34]; 3) observations of night-blindness within two weeks of administration of compounds that lower plasma vitamin A [35]; and 4) the observation that injected labeled vitamin A is rapidly incorporated into the neuroretina even in the dark [36].

The finding that the percent of deuterated vitamin A in the neuroretina was higher than the percent of deuterated vitamin A in the plasma and RE might reflect a contribution of plasma retinyl esters, which were nearly all (95%) deuterated, to the neuroretina vitamin A pool via retinal vessels, thus bypassing the RE. The delivery of retinyl esters via the chylomicron pathway has been proposed as an important mechanism to maintain retinal vitamin A homeostasis in addition to RBP-mediated vitamin A delivery [37-39]. Alternatively, the RE being a storage depot of vitamin A, may mimic the liver [40] and newly incorporated vitamin A might be first incorporated into the neuroretina for vision.

Like humans, swine are able to convert provitamin A carotenoids to vitamin A [41]. For example, 5 g of alfalfa meal is enough to supply the daily vitamin A requirement to a 200 kg pig [42]. In this study, we gave each swine, weighing between 7 and 17 kg, approximately 60 g of alfalfa meal per day, corresponding to 21 mg of carotene [43]. If our swine converted this 21 mg of carotene into vitamin A, then an additional 1.7 mg [44] of unlabeled vitamin A would have been added to each animal per day. This amount of unlabeled vitamin A is more than half the amount of the 3 mg of deuterated vitamin A that we administered and would significantly perturb the percentage of deuterated vitamin A in the eye. Thus, the finding of 95% enrichment in the retina suggests that dietary carotenoids did not significantly contribute to retinal vitamin A pools.

This study suggests that the majority of vitamin A in the plasma is newly ingested vitamin A, and supports the “last in first out” hypothesis of liver vitamin A usage. We further showed that, during a vitamin A adequate diet, plasma vitamin A is in fast equilibrium with retinal vitamin A pools. The findings suggest that retinal vitamin A can be rapidly swapped, within a few weeks, with a C20-D_3_-vitamin A drug to prevent vitamin A dimerization and suggest that the extent and rate of this swap can be estimated by measuring the ratio of C20-D_3_-retinol to retinol in the plasma.
